# Cannabigerol (CBG): A Comprehensive Review of Its Molecular Mechanisms and Therapeutic Potential

**DOI:** 10.3390/molecules29225471

**Published:** 2024-11-20

**Authors:** Shijia Li, Weini Li, Naseeb Kaur Malhi, Junwei Huang, Quanqi Li, Ziwei Zhou, Ruiheng Wang, Jiangling Peng, Tong Yin, Honggen Wang

**Affiliations:** 1 School of Biomedical and Pharmaceutical Sciences, Guangdong University of Technology, Guangzhou 510006, China; 2112112089@mail2.gdut.edu.cn (S.L.); 2112212009@mail2.gdut.edu.cn (J.H.); 2112312072@mail2.gdut.edu.cn (Q.L.); 3121008909@mail2.gdut.edu.cn (Z.Z.); 2 Department of Biomedical Science, Cedars-Sinai Medical Center, Cedars-Sinai Cancer Institute, Los Angeles, CA 90067, USA; weini.li@csmns.org (W.L.); ruiheng.wang@cshs.org (R.W.); 3 Department of Diabetes Complications and Metabolism, Arthur Riggs Diabetes and Metabolism Research Institute, Beckman Research Institute of City of Hope, Duarte, CA 91010, USA; nmalhi@coh.org; 4 South China Research Center for Acupuncture and Moxibustion, Medical College of Acu-Moxi and Rehabilitation, Guangzhou University of Chinese Medicine, Guangzhou 510006, China; 5 School of Pharmaceutical Sciences, Sun Yat-Sen University, Guangzhou 510006, China; wanghg3@mail.sysu.edu.cn

**Keywords:** Cannabigerol (CBG), endocannabinoid system, cannabinoid receptor, molecular mechanism

## Abstract

Cannabigerol (CBG), a non-psychoactive cannabinoid found in cannabis, has emerged as a promising therapeutic agent with a diverse range of potential applications. Unlike its well-known counterpart tetrahydrocannabinol (THC), CBG does not induce intoxication, making it an attractive option in the clinic. Recent research has shed light on CBG’s intriguing molecular mechanisms, highlighting its potential to modulate multiple physiological processes. This review delves into the current understanding of CBG’s molecular interactions and explores its therapeutic power to alleviate various conditions, including cancer, metabolic, pain, and inflammatory disorders, amongst others. We discuss how CBG interacts with the endocannabinoid system and other key signaling pathways, such as CB1, CB2, TPR channels, and α2-adrenoceptor, potentially influencing inflammation, pain, neurodegeneration, and other ailments. Additionally, we highlight the ongoing research efforts aimed at elucidating the full spectrum of CBG’s therapeutic potential and its safety profile in clinical settings. Through this comprehensive analysis, we aim to provide a deeper understanding of CBG’s role in promoting human health and pave the way for future research endeavors.

## 1. Introduction

The herbal use of Cannabis sativa plant extract has a long history of 5000 years [1]. In ancient China, the extracts from the Cannabis sativa plant were applied to treat gout, malaria, digestive disorders, muscle spasms, and pain [2]. Due to the psychoactive properties and potential for misuse or abuse, there have been decades-long debates over the legality of using cannabis as a therapeutic agent [1]. The psychoactive properties of cannabis are generated from one of its extracts, Delta-9-tetrahydrocannabinol (Δ-9-THC). Over time, with shifts in public perception and the generation of scientific evidence, cannabis use has been de-stigmatized, thus enhancing access to medicinal cannabinoid-based treatments. In 2018, Canada officially legalized cannabis for recreational and medical use, whilst in the same year, the US Drug Enforcement Administration removed hemp (defined as cannabis with less than 0.3% of Δ-9-THC) and hemp products from the controlled substances list. In 2021, Mexico legalized the recreational use of cannabis. Additionally, the recreational use of cannabis is legalized in Georgia, Luxembourg, Malta, South Africa, Thailand, and Uruguay. Moreover, the medical use of cannabis is legalized in more than fifty countries, including Australia, New Zealand, Brazil and others [3]. The US Food and Drug Administration (FDA) (2018) and the European Medicines Agency (EMA) (2019) approved cannabidiol (CBD) (Epidiolex^®^, UK) for the treatment of Dravet Syndrome (DS) and Lennox Gastaut syndrome (LGS) in patients 1 year of age and older. More recently, Epidiolex^®^ was approved for the treatment of tuberous sclerosis complex (TSC) by the FDA (2020) and EMA (2021) [4]. Sativex^®^ UK, an oral spray containing CBD and Δ-9-THC in a 1:1 ratio, is approved in several countries including the United Kingdom, the European Union (EU), and Canada for the treatment of multiple sclerosis-associated spasticity [5].

**Figure 1:**
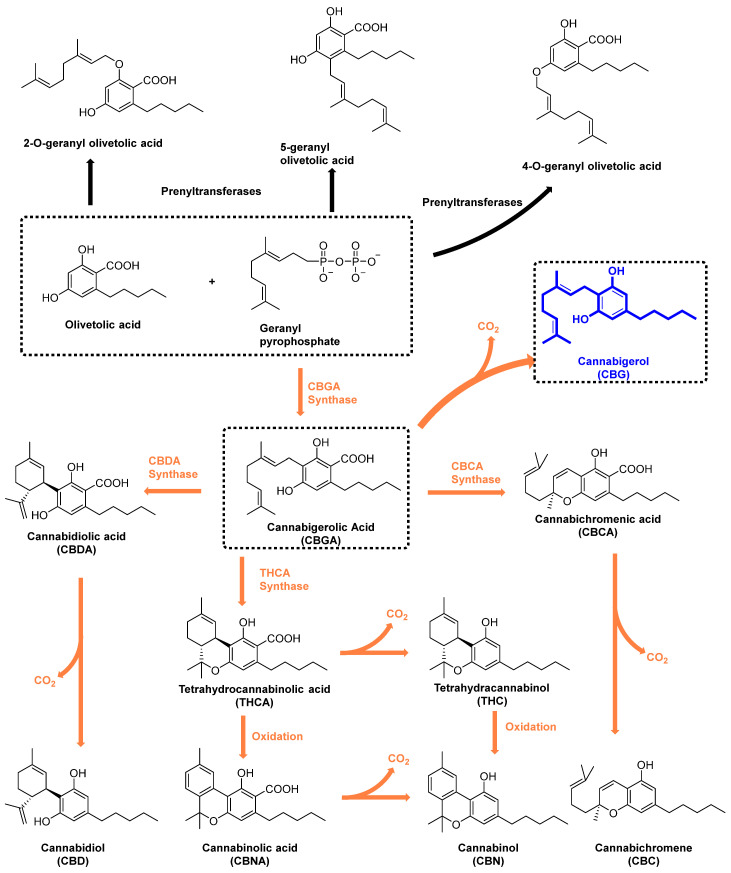
Chemical structures and biosynthesis pathways of the primary cannabinoids. Cannabinoid biosynthesis is initiated with the synthesis of CBGA from Geranyl pyrophosphate and Olivetolic acid. Geranyl pyrophosphate and Olivetolic acid served as substrates for 2-*O*-geranyl olivetolic acid, 5-geranyl olivetolic acid, and 4-*O*-geranyl olivetolic acid. CBGA is the primary precursor for most of the cannabinoids. CBDA synthase directs the conversion of CBGA to CBDA [23]. Decarboxylation of CBDA yields cannabidiol (CBD) [24]. Similarly, THCA synthase facilitates the formation of tetrahydrocannabinolic acid (THCA) from CBGA [25], with subsequent decarboxylation generating tetrahydrocannabinol (THC). Interestingly, THCA can also be oxidized to cannabinolic acid (CBNA), the precursor to cannabinol (CBN). Finally, CBCA synthase and decarboxylation lead to the formation of cannabichromene (CBC) through cannabichromenic acid (CBCA), in a similar fashion as CBD and THC [26].

**Figure 2:**
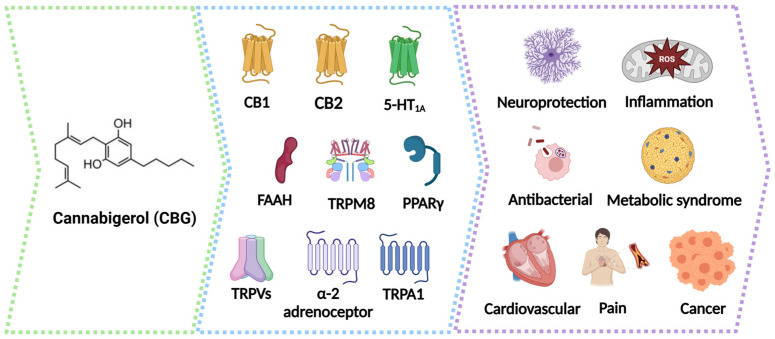
CBG-related receptors and potential therapeutical benefit. CBG acts as the agonist of the receptors of the TRPV family, PPARγ, and 5-HT_1A_, and as an antagonist of the receptor TRPVM8. CBG weakly agonizes the action of CB1 and partially acts as an agonist on CB2 receptor agonists. CBG also inhibits FAAH, which results in increased anandamide levels. Current studies revealed that CBG has potential therapeutic effects on neuroprotection, inflammation, antibacterials, metabolic syndrome, pain management, and cancer treatment. CB1, cannabinoid receptor type 1; CB2, cannabinoid receptor type 2; TRPM8, transient receptor potential cation channel 8; 5-HT_1A_, serotonin receptor 1A; FAAH, fatty acid amide hydrolase; TRPV, transient receptor potential vanilloid; PPARγ, peroxisome proliferator-activated receptor gamma.

Cannabigerol (CBG) is a non-psychoactive cannabinoid found in the cannabis plant. CBG converts from its acid form cannabigerolic acid (CBGA), which is the principal precursor to most of the other cannabinoids found in cannabis, such as Δ-9-THC, CBD, cannabinol (CBN), and cannabichromene (CBC) (Figure 1). CBGA is synthesized from geranyl diphosphate (GPP) and olivetolic acid under the presence of CBGA synthase. CBG is generated from CBGA by a non-enzymatic decarboxylation (Figure 1). In the presence of the respective enzymes (THCA synthase and CBDA synthase), the resulting acid products were converted to THC and CBD by decarboxylation, respectively [6]. While THC and CBD have been more widely studied than CBG, perhaps because they only make up 10% of the cannabinoid fraction in the cannabis plant, in recent years, CBG has had increased attention due to the wide range of potential health benefits, including neuroprotection [7], inflammation [8], antibacterials [9], metabolic syndrome [10], pain management [11], and cancer treatment [12] (Figure 2).

The therapeutic effect of the primary cannabinoid extracts largely relied on the effluence of the endocannabinoid system (ECS) [13]. The ECS is a complex network of receptors, enzymes, and molecules that help regulate various functions in the body, including mood, pain, appetite, memory, and sleep [14]. The ECS is a complex cell-signaling system. It consists of several key components: cannabinoid receptors (CB1 and CB2), signaling molecules called endocannabinoids (ECs) including anandamide (AEA) and 2-arachidonoylglycerol (2-AG), a transporter protein for AEA, and the enzymes responsible for the synthesis and degradation of ECs (fatty acid amide hydrolase, FAAH, or monoacylglycerol lipase, MGL) [13]. CB1 and CB2 are the two main types of cannabinoid receptors. Unlike THC and CBD, CBG does not bind directly to the CB1 receptors in the brain, which are responsible for the psychoactive effects of cannabis. Preliminary studies showed that CBG may be a partial agonist of CB1 and CB2 [15]. In 2012, a study demonstrated that CBG exhibited negligible binding affinities on CB1 and CB2 receptors in human cells [16]. Later studies revealed a more nuanced picture: CBG acts as a partial agonist for CB2 but has minimal direct activation of CB1. Interestingly, CBG may indirectly modulate CB1 by inhibiting the uptake of its natural ligand, AEA [17]. Beyond cannabinoid receptors, CBG interacts with other targets, including TRP channels, which play a crucial role in regulating the cytoplasmic calcium concentration from the extracellular sources as well as the calcium stored within the endoplasmic reticulum (ER). CBG serves as a strong agonist of TRPA1 and a weaker agonist of TRPV1, TRPV2, and TRPV4 while antagonizing TRPM8 (TRP melastatin type 8) [14,15,16]. Like ECs, CBG interacts with PPAR receptors as well [18]. Additionally, CBG reduces the activity of FAAH, the enzyme that breaks down anandamide. This elevates AEA levels and alters its effects on the body. However, it is important to note that CBG is less potent than CBD in inhibiting FAAH activity [19,20]. A single study performed by binding techniques in mouse brain membranes revealed that CBG behaves as a potent α2-adrenoceptor (α2AR) agonist and moderately potent 5-HT_1A_ receptor antagonist, which may explain its biological activity considering its slight affinity for cannabinoid receptors. It has been reported that CBG exerts 5-HT_1A_ receptor-mediated neuroprotective effects in vitro [21]. However, a recent study revealed that CBG hinders the inhibitory effect on the firing rate of NA-LC and 5-HT-DRN neurons produced by selective α2AR and 5-HT_1A_ receptor agonists, producing anxiolytic-like effects through the 5-HT_1A_ receptor [22]. In Table 1, the affinity and action of the CBG-related receptors are summarized. However, the underlying mechanisms for the effects of CBG remain largely elusive.

**Table 1:** Pharmacodynamic properties of CBG at related receptors.

Receptor	Affinity (nM)	Function	Reference
CB1	440–1045 (Ki)	Weak agonist	[15,16]
CB2	153.4–1225 (Ki)	Partial agonist	[15]
TRPA1	700	Agonist	[27,28]
TRPV1	1300	Agonist	[29,30]
TRPV2	1720	Agonist	[20,28]
TRPV3	1000	Agonist	[20,28]
TRPV4	5100	Agonist	[20,28]
TRPM8	160	Antagonist	[31,32]
α2AR	0.2–72.8	Agonist	[15]
5-HT_1A_	51.9	Antagonist	[21,22,33]
PPARγ	1270	Agonist	[16]

Abbreviations: CB1, cannabinoid receptor type 1; CB2, cannabinoid receptor type 2; TRPV1, transient receptor potential vanilloid type 1; TRPV2, transient receptor potential vanilloid type 2; TRPV3, transient receptor potential vanilloid type 3; TRPV4, transient receptor potential vanilloid type 4; TRPM8, transient receptor potential cation channel 8; α-2 adrenoceptor, alpha-2 (α2) adrenergic receptor; 5-HT1A, serotonin receptor 1A; PPARγ, peroxisome proliferator-activated receptor gamma.

There have been some excellent reviews on CBG, discussing its pharmacological potential [34], the bioactivity of CBD and its analogs [35], the biomedical relevance [36], and its interactions with voltage-gated sodium channels [37]. Differing from others, our review will discuss the molecular mechanisms of action of the therapeutic effects of CBG within different disease contexts.

## 2. Neuroprotection

**Figure 3:**
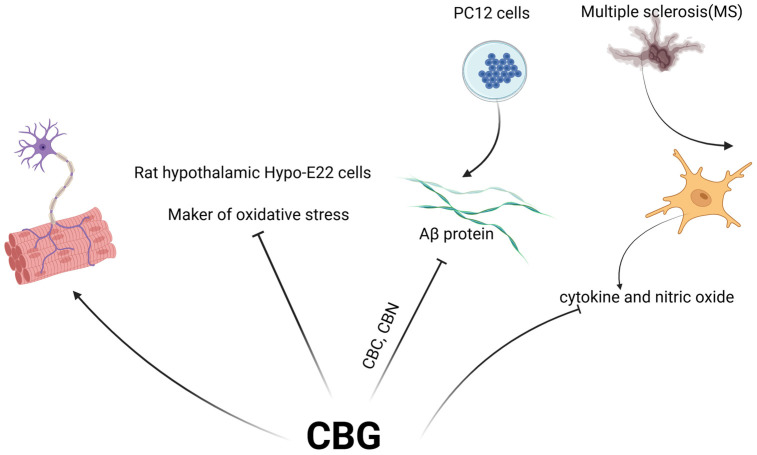
The neuroprotective effects of CBG. CBG treatment improved motor function and reduced a marker of oxidative stress in the hypothalamus. In combination with CBC and CBN, CBG significantly mitigated the toxic effects of Aβ protein in PC12 cells. Furthermore, CBG decreased cytokine and nitric oxide production associated with MS.

Neuroprotection is a therapeutic strategy aimed at safeguarding the nervous system. It encompasses a diverse range of approaches, all striving to achieve a common goal: shielding neurons from damage and promoting their survival [38]. CBG, like other cannabinoids, has shown potential neuroprotective properties, primarily through its interaction with the ECS. As discussed earlier, the ECS plays a crucial role in regulating various physiological processes, including those related to the nervous system. CBG may help reduce neuroinflammation, which is associated with various neurodegenerative conditions, such as Alzheimer’s disease, Parkinson’s disease, and multiple sclerosis (MS). A preliminary study explored the potential neuroprotective effects of CBG in two mouse models of Huntington’s disease [7]. In this study, CBG treatment improved motor function in mice with Huntington’s disease and partially normalized the expression of genes linked to Huntington’s disease in mice [7]. Oxidative stress is a significant factor in neurodegenerative diseases, and antioxidants can help protect neurons from damage caused by free radicals. In 2020, a study investigated the neuroprotective properties of CBG, as well as CBD, in rat hypothalamus cells (Hypo-E22 cells) and tissue. The results suggest that CBG decreased a marker of oxidative stress in the hypothalamus, potentially protecting cells from damage [39]. Amyloid beta (Aβ) accumulation is a hallmark of Alzheimer’s disease and is linked to neuronal death. Smid and coworkers revealed that CBG, along with CBC and CBN, significantly inhibited the toxic effects of Aβ protein in PC12 cells, a common model for studying neurodegeneration. These results support CBG’s ability to maintain healthy cell morphology and suggest a potential neuroprotective effect. However, the exact mechanisms by which CBG protects against Aβ toxicity were not investigated in this study [40]. Fleisher-Berkovich et al. investigated the potential of CBG for MS treatment by focusing on its ability to modulate microglial activation. Microglia are the primary immune cells of the brain, but their overactivation can contribute to neurodegeneration in MS (Figure 3). This pre-clinical study indicated that CBG reduced MS-induced cytokine and nitric oxide production in the in vitro and in vivo setting, slowing disease progression and thus could be a potential therapeutic strategy [41].

Overall, current studies suggest that CBG has promise as a neuroprotective agent, but the underlying mechanisms warrant further investigation, as well as whether the results translate to a human setting. Furthermore, studying CBG independently from other cannabinoids could help pinpoint distinct therapeutic properties and specific mechanisms in the context of the central nervous system.

## 3. Anti-Inflammatory

Various studies have suggested the use of cannabinoids as possible treatments for inflammatory diseases [42], such as inflammatory bowel disease (IBD) [43], liver injury [44], rheumatoid arthritis [45], and allergic asthma [46]. Both CB1 and CB2 receptors have been found on immune cells suggesting that cannabinoids play an important role in immune regulation. Recent studies showed that cannabinoids downregulate cytokine and chemokine production and, in some models, upregulate T-regulatory cells (Tregs) as a mechanism to suppress inflammatory responses [42]. Increasing evidence indicated that CBG modulates the pro-inflammatory proteins by activating cannabinoid receptors CB1 and CB2 [8,47,48].

Inflammatory bowel disease (IBD) is a group of chronic inflammatory conditions of the gastrointestinal tract. The two primary types of IBD are Crohn’s disease and ulcerative colitis. IBD is characterized by periods of remission and exacerbation [49]. The exact cause of IBD remains unknown, but it is believed to involve a complex interplay of genetic, environmental, and immune factors [43]. In a murine model of colitis, induced by dinitrobenzene sulphonic acid, CBG reduced nitric oxide production in macrophages, mitigating the colitis phenotype. Interestingly, only the action of CBG on the CB2, but not the CB1 receptor, was key to the attenuation. Additionally, CBG exhibited a noteworthy capacity to diminish colitis-induced reactive oxygen species (ROS) formation in intestinal epithelial cells [8]. The effect of CBG was associated with modulation of cytokine (IL-1b, IL-10, and interferon-γ) levels and down-regulation of iNOS (but not COX-2) expression. These promising outcomes suggest that CBG merits consideration for further clinical experimentation as a potential therapeutic agent for patients with IBD [8].

**Figure 4:**
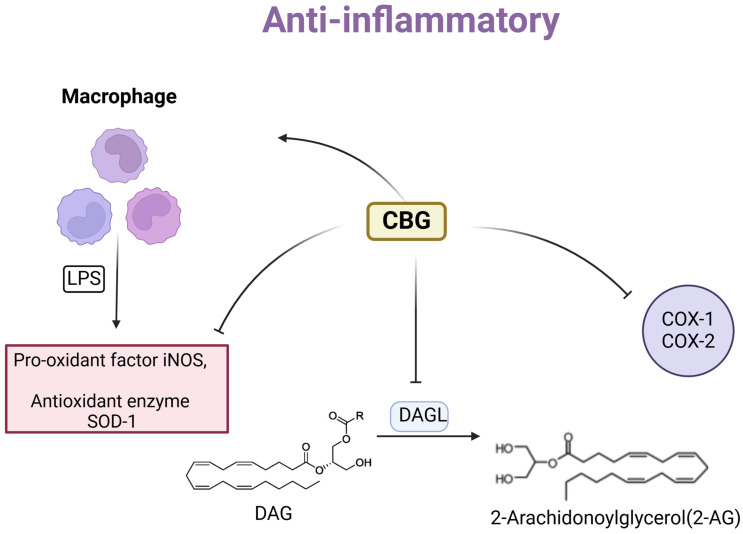
The mechanism of CBG anti-inflammatory effects. The figure depicts the main mechanisms by which CBG affects inflammation and antioxidant activity. CBG reduces the activity of inducible nitric oxide synthase (iNOS) and modulates the expression of superoxide dismutase-1 (SOD-1). Furthermore, CBG inhibits diacylglycerol lipase (DAGL) and decreases the activity of cyclooxygenase-1 (COX-1) and cyclooxygenase-2 (COX-2) enzymes.

CBG appears to regulate redox balance through multiple mechanisms. As mentioned earlier, it reduces the activity of iNOS, a major pro-oxidant factor [8,50]. One study identified the antioxidant effect of CBG in countering hydrogen peroxide (H_2_O_2_)-induced oxidative stress in murine RAW264.7 macrophages. The findings demonstrated that 10 μM of CBG alleviated oxidative stress through the activation of CB2R. This was evidenced by the observation that the protective effects of CBG on H_2_O_2_-stimulated macrophages were negated when the CB2R antagonist AM630 was used [51]. Additionally, CBG activates PPARγ and modulates the expression of SOD-1, an antioxidant enzyme typically suppressed by pro-inflammatory factors. By influencing these pathways, CBG helps maintain a healthy cellular balance between oxidants and antioxidants [50]. CBG inhibits diacylglycerol lipase (DAGL), an enzyme that creates the endocannabinoid 2-AG. Whilst the expression of COX-2 was previously found to be unchanged by CBG [8], here they found CBG reduced the activity of COX-2 (as well as COX-1) enzymes. This altered the levels of both endocannabinoids and other lipid mediators, affecting cell signaling and potentially impacting inflammation and antioxidant activity (Figure 4) [52]. NF-κB is a dimeric transcription factor that is typically sequestered in the cytoplasm by inhibitory IκB proteins. Upon stimulation, such as oxidative stress, IκB is phosphorylated and degraded, allowing NF-κB to translocate to the nucleus and induce gene expression. CBG treatment significantly increased cytoplasmic IκB-α staining and reduced nuclear NF-κB staining in H2O2-stimulated cells [53]. One paper reported that CBG was rapidly metabolized by human cytochrome P450 enzymes into bioactive oxidized metabolites. Both CBG and its oxidized metabolites were shown to effectively reduce inflammation in LPS-stimulated BV2 microglial cells [54].

Notably, CBG surpasses the efficacy of vitamin C in reducing ROS levels in human dermal fibroblasts (HDF) [55] and exhibits anti-inflammatory properties in skin cells. A key clinical study highlights the skin health-boosting attributes of CBG, where a 0.1% CBG serum yielded statistically significant improvements in transepidermal water loss and the appearance of redness following sodium lauryl sulfate-induced irritation [47]. Moreover, CBG effectively reduced pro-inflammatory proteins, including 4-hydroxynonenal protein adducts induced by UV-induced keratinocyte damage, and elevated the levels of proteins associated with cellular locomotion [55]. These compelling findings emphasize CBG’s promising role in promoting skin well-being and warrant its continued exploration for skincare applications.

Hepatic inflammation is a primary pathology that can lead to hepatic steatosis and fibrosis in non-alcoholic steatohepatitis (NASH) patients [56,57]. Aljobaily and coworkers reported that a low dose (low dose: 2.46 mg/kg/day, high dose: 24.6 mg/kg/day) of CBG exhibited significant efficacy in alleviating hepatic and inflammation in methionine/choline-deficient (MCD)-induced NASH. Moreover, mice undergoing this treatment exhibited decreased hepatic ballooning and leukocyte infiltration, with decreased expression of both CB1 and CB2. However, a high dose of CBG administration actually increased inflammation and fibrosis and enhanced liver damage [58]. As such, this study provides a platform for future (pre-)clinical work, which should focus on elucidating the correct dosage and underlying factors of the dose-dependent response.

## 4. Antibacterial Effect

The emergence of antibiotic-resistant bacteria poses a significant threat to global health [59]. The continuous development of novel antibacterial agents is crucial to combat this growing public health concern [60]. CBD and terpene have been reported to have antibacterial activity. Despite good to moderate activity against specific bacteria like *Enterococcus* and *Salmonella*, the effect was weaker against a broader range [61]. CBG has garnered increasing interest for its potential antibacterial properties, and exhibits a broad spectrum of antibacterial activity, particularly against Gram-positive bacteria, unlike its counterparts [9,62,63]. Studies highlight its effectiveness against common pathogens like Staphylococcus aureus (including methicillin-resistant strains, MRSA) and *Streptococcus mutans*, a bacterium associated with dental cavities [9,63,64]. Interestingly, CBG also demonstrates activity against some Gram-negative bacteria like *Vibrio harveyi*, suggesting its potential for a wider range of infections [64].

The exact mechanisms underlying CBG’s antibacterial effects remain under investigation. Current studies revealed that CBG may disrupt the bacterial cell membrane, a crucial barrier to survival. This disruption can lead to the leakage of essential cellular components and ultimately cell death [62,63]. Furthermore, CBG has been shown to inhibit biofilm formation in *S. mutans* and *V. harveyi* [63,64]. Biofilms are communities of bacteria encased in a protective matrix that makes them highly resistant to antibiotics [65]. This could be a valuable strategy as biofilms are implicated in chronic infections. Bacteria often communicate through a process called quorum sensing. Quorum sensing is crucial in various bacterial activities, including virulence and biofilm formation. In the context of virulence, quorum sensing enables bacteria to coordinate the production and release of toxins and other virulence factors. This synchronized attack can overwhelm the host’s defenses, leading to severe infections. Additionally, quorum sensing facilitates biofilm formation, a complex community of bacteria encased in a self-produced extracellular matrix [66]. CBG may interfere with this communication, hindering the bacteria’s ability to coordinate activities like biofilm formation and virulence [67].

These studies provide a strong foundation for further research on CBG’s antibacterial properties. Research on CBG’s antibacterial properties is still in its early stages. Further studies of the antibacterial properties of CBG should focus on the following key areas: (1) Translating these in vitro findings to in vivo, (2) CBG-specific molecular interactions with bacterial targets, (3) Exploration of synergistic effects between CBG and conventional antibiotics. This will enable targeted drug development in patients and help combat the concerning rise in antibiotic resistance. In conclusion, CBG emerges as a promising candidate in the fight against bacteria. Its broad spectrum of activity, biofilm inhibition capabilities, and potential for synergy with conventional antibiotics warrant further investigation. As research progresses, CBG may pave the way for novel therapeutic strategies to combat bacterial infections and contribute to the effort against antibiotic resistance.

## 5. Hypotension/Vasoconstriction

Several studies have shown CBD’s beneficial effects on the cardiovascular system [68] by interacting with various aforementioned receptors, including CB1, CB2, TRPV1, PPARs, and 5-HT_1A_ [69]. The effects of CBG on the cardiovascular system, particularly blood pressure, are largely under investigation. CBG acts as a weak or partial agonist at CB1 and CB2 receptors and exhibits nanomolar affinity to α2AR in peripheral tissues and the central nervous system [15,70]. Activation of α2AR inhibits the release of norepinephrine causing vasodilation (relaxation of blood vessels), bradycardia (slower heart rate), and decreased contractility (weaker heart contractions), ultimately resulting in lower blood pressure [71]. Accordingly, Arnold and coworkers demonstrated a significant reduction in blood pressure in mice following CBG treatment. In phenotypically normal mice, acute CBG (10 mg/kg, intraperitoneal injection) significantly lowered mean blood pressure with no apparent dose responsiveness. However, at this dose, the depressor effect of CBG was not as effective as Guanfacine (α2AR agonist) [72]. Importantly, a following abstract published by the same group revealed that chronic CBG administration (10 mg/kg) produced a significant decrease in blood pressure in phenotypically normal male mice [73].

The precise mechanisms by which CBG lowers blood pressure remain unclear, but the provided research offers some clues. The study conducted by Arnold and coworkers suggests that similar to the brain, CBG interacts with α2AR [72], a class of receptors known to play a role in blood pressure regulation. Activation of these receptors can lead to blood vessel constriction (vasoconstriction), ultimately reducing blood pressure. This hypothesis is supported by the observation that the hypotensive effect of CBG was blocked by pretreatment with an α2AR antagonist. While the α2AR pathway appears to be a key contributor, other mechanisms may be involved. Additionally, CBG has a role in pain management [11], which can be indirectly linked to blood pressure. Chronic pain can lead to elevated blood pressure, and CBG’s pain-relieving properties might contribute to overall blood pressure regulation. However, this connection needs further exploration.

Glaucoma is a primary cause of irreversible vision loss, and intraocular pressure (IOP) remains the only modifiable risk factor for disease progression [74]. While both CBD and THC have demonstrated IOP-lowering effects in glaucoma patients [75], the therapeutic potential of CBG warrants further investigation. Preclinical studies in felines have indicated that CBG effectively reduces IOP, with a more pronounced hypotensive effect observed following chronic administration [51]. Notably, CBG did not induce ocular or neurotoxic adverse effects in rodent models, as observed with other cannabinoids [76]. Given the neurodegenerative nature of glaucoma, characterized by the progressive loss of retinal ganglion cells, the potential neuroprotective properties of CBG may offer additional therapeutic benefits.

Whilst the current literature is promising, further research is needed to explore additional pathways, such as modulation of ion channels or interactions with the ECS, that could influence blood pressure. Moreover, the use of relevant animal models such as Ldlr knockout mice, and eventually human clinical trials is crucial to understanding the translational potential of CBG.

## 6. Treatment of Cancer

Accumulating evidence has substantiated the anti-tumorigenic properties of CBG, showcasing its efficacy in reducing cell proliferation, migration, and survival in various tumor types, including prostate cancer, glioblastoma, colorectal carcinoma, pancreatic cancer, and breast cancer.

Cannabinoids have been well-researched in cancer, with the bulk of the research focused on THC and CBD, both of which increase the survival of glioblastoma patients [77,78]. These two agents have progressed to clinical trials, coupling chemotherapy with phytocannabinoids to assess breast and colon cancer patient recovery. CBG, whilst not at the clinical trial stage as of yet, has shown much promise with regard to treating glioblastoma. CBG, like many other cannabinoids, is lipid-soluble. This property allows it to easily pass through the lipid bilayer of cell membranes, including the blood–brain barrier [79], and it was found to reduce C6-rat glioma cell proliferation [80]. A study in 2021 found that CBG alone and in combination with CBD effectively promoted apoptosis in glioblastoma stem cells [12]. CBG inhibited the invasive behavior of glioblastoma cells, in a similar way as CBD combined with temozolomide, a chemotherapeutic. The CBG plus CBD combination was found to be more effective than CBD with THC in inducing cytotoxicity and reducing invasion in a 3D spheroid assay [12]. The inhibitory effects of CBG and CBD on glioblastoma stem cells are likely primarily involved in the modulation of GPR55 and TRPV1 signaling [30]. As solid tumors progress, the tumor microenvironment (TME) undergoes changes that transform into a highly immunosuppressive milieu. Regulatory myeloid cells play key roles in the immunosuppressive environment and activate tumor-secreted cytokines. It was found that CBG decreased the CSF-1 secretion by melanoma cells. CBG significantly reduces the expansion and macrophage transition of the monocytic-myeloid-derived suppressor cells (MDSCs) subpopulation. It is a promising treatment for tumors with elevated CSF-1 expression [81]. In a study of minor phytocannabinoids on Multiple myeloma (MM), CBG was found to inhibit MM cell growth in a dose-dependent manner and reduce the invasion of MM cells toward osteoblasts cells [82].

During tumor progression, genetic and epigenetic alterations can enable tumors to evade immune detection, a phenomenon known as immune escape [83]. The adaptive immune system’s ability to detect and eliminate emerging tumors relies on immune surveillance by cytotoxic T lymphocytes (CTLs). CTLs recognize tumor cells by binding to major histocompatibility complex class I (MHC-I) molecules, which present fragments of tumor-derived proteins [84]. Dada et al. demonstrated that CBG can reverse this immune escape phenotype in vitro by upregulating MHC-I expression on various metastatic tumor cells, thereby rendering them susceptible to T cell recognition [85].

**Figure 5:**
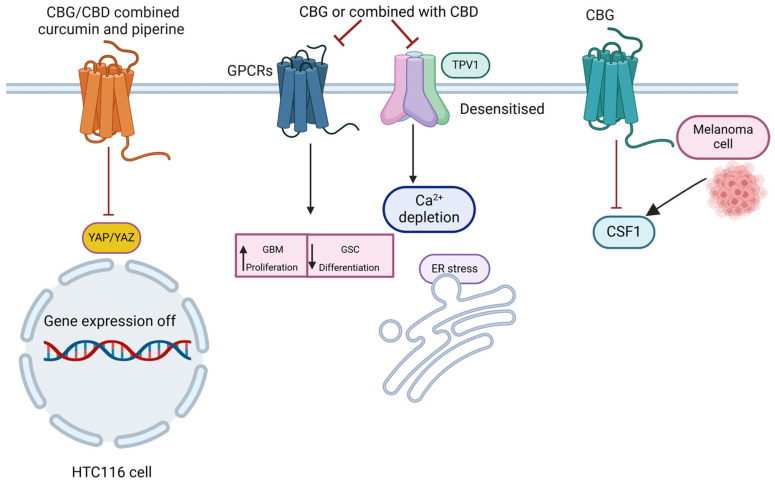
The primary pathways of CBG’s anti-tumor effects. The figure illustrates the principal signaling pathways. The combined use of CBG/CBD, curcumin, and piperine leads to a reduction in YAP expression levels, thereby regulating the Hippo pathway. CBD/CBG also inactivates GPR55 signaling, resulting in decreased GBM proliferation and promoting cell differentiation. In addition, CBD/CBG desensitized TRPV1, inducing a pro-apoptotic response via Ca^2+^ depletion and triggering ER stress. Moreover, CBG reduced CSF-1 secretion by melanoma cells [81].

The mechanism of anti-tumor effects of CBG involved the Hippo-YAP, TRP channels, EGFR-RAS pathways, and targeted tumor-secreted cytokines (Figure 5). The Hippo-YAP pathway is a crucial cellular signaling pathway that regulates organ size, cell proliferation, and apoptosis. When this pathway is dysregulated, it can contribute to tumorigenesis [86]. Recently, a paper evaluated the cytotoxic and cytostatic effects of CBG alone or in combination with curcumin and piperine in colon carcinoma cell lines HCT116 and HT29 [87]. Both mono and combination treatments demonstrated a notable reduction in the expression of the YAP oncogene in cell lines. Cannabinoid compounds, along with curcumin/piperine, effectively inhibit the proliferation of HCT116 cells by activating the Hippo signaling pathway. In contrast, the suppression of proliferation in the HT29 cell line appears to be mediated by a decrease in YAP expression independent of Hippo signaling [87]. These findings underscore CBG’s multifaceted potential in modulating critical pathways, presenting it as a noteworthy candidate for therapeutic interventions in various cancers. In a study conducted by Lamtha and coworkers, the kinase-inhibition activity of CBG to tyrosine-kinase of EGFR, which is involved in over 70% of all cancers, was evaluated. The binding kinetics information indicated that CBG had a high binding affinity and kinase inhibitory activity against EGFR-TK. In addition, CBG significantly induced cell apoptosis in the EGFR-positive cancer A431 line [88]. Zeppa et al. investigated the effects of CBG on pancreatic ductal adenocarcinoma (PDAC) cell lines PANC-1 and MIAPaCa-1. Their findings demonstrated that CBG reduced mTOR protein expression and suppressed EGFR expression in both of the two cell lines [89]. Moreover, the data supported that CBG reduces downstream RAS signaling.

The combination of CBG and conventional chemotherapy agents holds promise for enhancing treatment efficacy. Potentiation of chemotherapy and reduction of chemotherapy side effects are the potential ways by combining CBG with chemotherapy. CBG alone was found to suppress hormone-refractory prostate cancer (HRPC) development in a mouse model of prostate cancer (TRAMP mice) via reprogramming metabolic and oncogenic signaling, although this effect was less pronounced than CBD. However, the two in combination exhibited potent anti-cancer effects even in mice unresponsive to enzalutamide (androgen receptor antagonist) treatment. As such, combination therapy may be an attractive adjuvant therapeutic option for prostate cancer patients [90].

Further studies in pre-clinical in vivo models on CBG are necessary to better understand its role in cancer progression and immune escape. In vivo studies would be particularly useful to more deeply investigate its anticancer effects More research is needed to fully understand the interactions between CBG and chemotherapy drugs. However, the potential benefits of this combination warrant further investigation.

## 7. Metabolic Syndrome

Metabolic syndrome is a group of abnormalities that increase the risk of the development of cardiovascular disease, liver disease, and type 2 diabetes. The diagnosed criteria of metabolic syndrome include abdominal obesity, hypertension, hyperglycemia, low high-density lipoprotein (HDL) cholesterol levels, and high triglyceride levels.

Recent studies on CBG provide a potential pharmacotherapy strategy for metabolic syndrome. Notably, CBG is the sole known cannabinoid that activates the adrenergic receptor. Additionally, as mentioned earlier, CBG and its derivatives have efficacy in modulating PPARγ receptors. In 2019, in vitro modeling of phytocannabinoids suggested CBG as a dual PPARα/γ agonist [91]. A follow-up study in 2020 reported that CBG’s role as a PPARα/γ agonist promoted the maturation of bone marrow-derived mesenchymal stem cells into adipocytes [92]. Additionally, CBG has been shown to reduce appetite and induce weight loss by blocking CB1 receptors. Additionally, CBG has been shown to increase the activity of brown adipose tissue (BAT), which is responsible for burning calories and generating heat. HUM-234, a derivative of CBG, has been shown to prevent high-fat diet-induced weight gain and lower serum levels of liver enzymes ALT and AST [10]. Consequently, CBG emerges as a promising candidate for therapeutic exploration in combating this debilitating condition. The potential of CBG to stimulate adipogenesis and improve insulin sensitivity implies a prospective avenue for enhancing metabolism, fostering more efficient calorie burning, and facilitating weight loss.

## 8. Pain Management

Pain is a complex phenomenon that extends beyond a simple physical sensation. It encompasses a blend of sensory-physiological, motivational-affective, and cognitive-evaluative components, shaping our overall experience of discomfort. Nociceptive pain arises from direct damage to body tissues and is often described as sharp, aching, or throbbing. It serves as a warning signal, alerting us to potential injury [93]. Inflammatory pain stems from noxious stimuli associated with the body’s inflammatory or immune response. It is often accompanied by swelling, redness, and heat in the affected area [94]. Chronic pain is defined as pain lasting longer than 3 months; it can be recurrent or constant, significantly impacting a person’s daily life and causing physical and emotional distress [95]. Neuropathic pain results from damage to sensory or spinal nerves, leading to distorted pain messages sent to the brain [96]. This can manifest as burning, tingling, or shooting pains. Neuropathic pain affects 7–10% of the population and is caused by a lesion or disease of the somatosensory nervous system [97].

Cannabinoids have shown promise, in preclinical models, in reducing pain alone or in combination with other cannabinoids. In 2022, Graziane and coworkers reported that CBG treatment significantly reduced pain sensitivity in mice with chemotherapy-induced peripheral neuropathy. CBG’s pain-reducing effects seem to be mediated by its interaction with specific receptors, including α2AR, CB1, and CB2 [11]. The work reported by Ghovanloo et al. suggested that CBG reduces the excitability of the neurons in the dorsal root ganglion, a region involved in pain signal transmission, by inhibiting sodium conductance [98]. Consequentially, CBG might make it harder for pain signals to be transmitted effectively. This research unveils a potential mechanism for CBG’s indirect neuroprotective effects. By reducing pain signaling in the nervous system, CBG might offer relief from chronic pain conditions. This finding adds to the growing body of evidence suggesting CBG’s potential role in protecting the nervous system by managing pain.

## 9. Summary and Future Research Directions

CBG has emerged as a potential therapeutic agent with a diverse range of effects. Although research on CBG is still in its early stages, its unique molecular mechanism and promising therapeutic profile warrant further exploration. CBG exhibits similar activity and affinity characteristics to Δ9-THC and CBD on cannabinoid receptors but also has a unique affinity for other receptors, such as the α2AR and 5-HT_1A_ receptors. CBG’s diverse mechanisms translate into a wide range of potential therapeutic applications, including neuroprotection, anti-inflammation, antibacterial properties, hypotension, cancer treatment, pain management, and metabolic syndrome. Additionally, some CBG users report improved sleep, although this has not yet been confirmed by clinical studies. Like other cannabinoids, CBG has been shown to have beneficial effects in glaucoma patients by reducing intraocular pressure, a primary risk factor for glaucoma. However, the exact mechanisms and long-term effects are yet to be fully understood.

In conclusion, CBG presents as a promising therapeutic agent with a unique molecular profile and a broad spectrum of potential benefits. As research progresses, CBG has the potential to become a valuable addition to the therapeutic arsenal for a range of conditions. Ongoing and future studies will likely uncover more about its efficacy, safety, and optimal usage parameters, leading to more precise applications in clinical settings. Further research focusing on clinical trials, detailed mechanistic studies, and optimized delivery systems is crucial to unlocking the full potential of CBG for various medical applications. Additionally, exploring its synergy with other cannabinoids and traditional medications could open new avenues for combination therapies. As we deepen our understanding of CBG, it may lead to breakthroughs in treating complex conditions, ultimately improving patient outcomes and expanding the scope of cannabinoid-based medicine. The next decade could see CBG integrated into mainstream medical practice, revolutionizing the approach to many chronic and debilitating diseases.

## Data Availability

No new data were created in this study.

## Abbreviations

**Abbreviations**	**Full name**
2-AG	2-arachidonoylglycerol
5-HT_1A_	serotonin receptor 1A
AEA	anandamide
Aβ	amyloid beta
CB1	cannabinoid receptor type 1
CB2	cannabinoid receptor type 2
CBC	cannabichromene
CBCA	canabichromenic acid
CBD	cannabidio
CBDA	cannabidiolic acid
CBG	Cannabigerol
CBGA	Cannabigerolic acid
CBN	Cannabinol
CBNA	Cannabinolic acid
COX-1	Cyclooxygenase-1
COX-2	Cyclooxygenase-2
Δ-9-THC	Delta-9-tetrahydrocannabinol
DAGL	diacylglycerol lipase
DS	Dravet Syndrome
ECS	Endocannabinoid system
ECs	Endocannabinoids
EMA	European Medicines Agency
ER	endoplasmic reticulum
EU	European Union
FAAH	Fatty acid amide hydrolase
FDA	Food and Drug Administration
GPP	Geranyl diphosphate
HDF	Human dermal fibroblasts
HDL	High-density lipoprotein
HRPC	Hormone-refractory prostate cancer
IBD	Inflammatory bowel disease
iNOS	Nitric oxide synthase
IOP	Intraocular pressure
LGS	Lennox Gastaut Syndrome
MCD	Methionine/choline-deficient
MDSCs	Monocytic-myeloid-derived suppressor cells
MGL	Monoacylglycerol lipase
MRSA	Methicillin-resistant strains
MS	Multiple sclerosis
NASH	Non-alcoholic steatohepatitis
PPAR	Peroxisome proliferator-activated receptor
ROS	Reactive oxygen species
SOD-1	Superoxide dismutase-1
THC	Tetrahydrocannabinol
THCA	Tetrahydrocannabinolic acid
TME	Tumor microenvironment
Tregs	T-regulatory cells
TRPM8	transient receptor potential cation channel 8
TRPM8	TRP melastatin type 8
TRPV	transient receptor potential vanilloid
TSC	Tuberous Sclerosis Complex
α2AR	α2-adrenoceptor
2-AG	2-arachidonoylglycerol
